# Whitening Technique Based on Gram–Schmidt Orthogonalization for Motor Imagery Classification of Brain–Computer Interface Applications

**DOI:** 10.3390/s22166042

**Published:** 2022-08-12

**Authors:** Hojong Choi, Junghun Park, Yeon-Mo Yang

**Affiliations:** 1 Department of Electronic Engineering, Gachon University, Seongnam 13306, Korea; 2 School of Electronic Engineering, Kumoh National Institute of Technology, Gumi 39177, Korea

**Keywords:** whitening technique, Gram–Schmidt orthogonalization, motor imagery classification, eigenface analysis

## Abstract

A novel whitening technique for motor imagery (MI) classification is proposed to reduce the accuracy variance of brain–computer interfaces (BCIs). This method is intended to improve the electroencephalogram eigenface analysis performance for the MI classification of BCIs. In BCI classification, the variance of the accuracy among subjects is sensitive to the accuracy itself for superior classification results. Hence, with the help of Gram–Schmidt orthogonalization, we propose a BCI channel whitening (BCICW) scheme to minimize the variance among subjects. The newly proposed BCICW method improved the variance of the MI classification in real data. To validate and verify the proposed scheme, we performed an experiment on the BCI competition 3 dataset IIIa (D3D3a) and the BCI competition 4 dataset IIa (D4D2a) using the MATLAB simulation tool. The variance data when using the proposed BCICW method based on Gram–Schmidt orthogonalization was much lower (11.21) than that when using the EFA method (58.33) for D3D3a and decreased from (17.48) to (9.38) for D4D2a. Therefore, the proposed method could be effective for MI classification of BCI applications.

## 1. Introduction

The human brain is composed of several encephalic regions that can control and record various human activities, such as movement, memory, and emotions [1,2]. In a broad sense, in a brain–computer interface (BCI), there are two types of categories available in the field of technology. One is unidirectional BCIs and the other is bidirectional BCIs. In the unidirectional BCI, the BCI is used to generate the pathway or channel for communication and control of other human parts or external devices using the brain without motor neuron intervention, such as the tongue and hand [3,4,5]. A BCI system can be broadly classified into three parts, namely the signal acquisition, signal processing, and application interface [6]. The signal processing is divided into three further parts: preprocessing, feature extraction, and classification [6]. The signal acquisition method for a BCI system is generally electroencephalogram (EEG) [7], which is used to measure the electrical signals that are generated from the human brain to estimate human activities. Unprocessed EEG is known as raw EEG, which undergoes a signal processing process for classification such as signal selection, filtering, and feature extraction [8]. An application interface such as the BCI system is controlled by classified features. The classification is a type of final stage to categorize to which class the features belong. In a BCI system, a human has a thought with intention and simulates physical actions, which corresponds to the scope of motor imagery (MI) classification problems. Therefore, MI classification has been studied for EEG analysis and classification because it can exhibit unknown EEG data that are generated by thoughts via moving human body parts, such as the hands, feet, and tongue [9].

The brain–computer interfaces (BCI) are one of the human machine interfaces (HMI) or human communication systems, which enable users to send commands to computers by using brain activity only. The potential of these activities is generally measured by EEG under 10-20 systems [3]. The BCI is generally designed according to a pattern recognition approach, i.e., by extracting features from raw EEG signals and using a discrete classifier to identify the user mental state from such derived features from raw data [10]. The previously proposed eigenface analysis (EFA) algorithm is a feature extraction method from raw EEG data which builds up neuro images emphasizing the discriminability of classes, and the feature is a determinate tool including accuracy.

Among the classification schemes, the linear classification method known as linear discriminant analysis (LDA) is used extensively in MI classification [1,10,11,12,13]. LDA is used to maximize two class variances using the Gaussian method. Furthermore, the support vector machine is a statistical method used in MI classification [14].

In a statistical signal processing, whitening transform is aimed to provide a unit variance and a minimum covariance for the given random data; hence, the covariance matrix is an identity matrix [15,16,17]. In the 10-20 systems of BCI applications, minimizing the dependency between experimental participants or subjects is an essential and key factor to solve classification problems. Furthermore, it would be essential to reduce the original correlation of signals between electrode channels [18].

Differences between features and classes in BCI-features refer to an important quality or ability of BCI signals whereas classes of BCI refer to unique physical activities that make MI signals distinguishable. Features are abstractive, and classes are concrete in classification problems [19].

**Figure 1:**
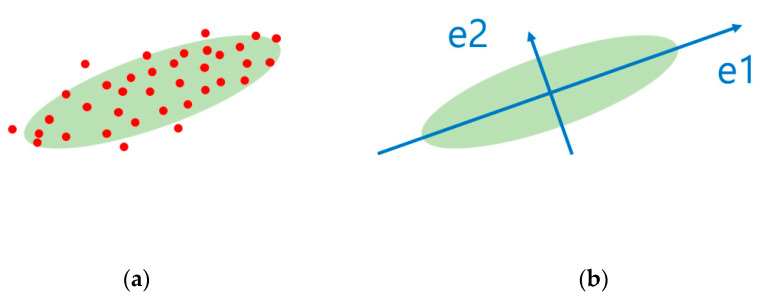
(**a**) Original distributed data and (**b**) distributed data with PCA technique applied.

In principal component analysis (PCA), there are *n* numbers of principal components for an n-dimensional data. Each principal component represents a direction vector with the direction of the largest data variance or eigenvalues. In Figure 1, the vectors e1 and e2 indicate the largest and next largest data variance of variance among ‘*n*’ eigenvalues, respectively. Because the covariance matrix of PCA is symmetric, the principal components are orthogonal and uncorrelated with one another. That is, the determination of the principal component can be used for analysis in a direction that shows the distribution shape effectively and can reduce the dimensions with only the main components. Therefore, PCA can be used for feature selection and dimension reduction because it can easily identify the representative data pattern.

PCA is a method for reducing the dimensions to identify the principal components from distributed data [20]. The PCA technique is generated from the geometric optimization problem to determine the hyper-plane that is the most appropriate for classifying the data distribution in n-dimensional space [5,21]. It was developed to identify the principal components that maximize the original variable variances [22]. Figure 1 presents distributed data using the PCA technique [23].

As illustrated in Figure 1, there are n principal components for an n-dimensional data distribution. Each principal component represents a direction vector with the direction of the largest data variance. In Figure 1, the vectors e1 and e2 indicate the largest and next largest data variance, respectively. Moreover, the principal components are orthogonal and uncorrelated with one another. That is, the determination of the principal component can be used for analysis in a direction that shows the distribution shape effectively and can reduce the dimensions with only the main components. Therefore, PCA is used for feature selection and dimension reduction because it can easily identify the representative data pattern. The covariance of the PCA calculation is presented below:

(1) $$Cov\;\left[X,Y\right]=E\left[\left(X-\bar{X}\right)\left(Y-\bar{Y}\right)\right]=\sum^{\;}\frac{\left(X-\bar{X}\right)\left(Y-\bar{Y}\right)}{n}$$

where *X* and *Y* are unknown variables, *Cov* [*X*, *Y*] is the covariance matrix of *X* and *Y*, and n is the number of data.

The covariance matrix can be calculated as an *n* × *n* matrix for *n* data. 

Whitening or whitening transform is a preprocessing scheme that applies PCA. In this study, we propose the BCI channel whitening (BCICW) scheme to improve the classification by minimizing the variance of the MI classification accuracy for BCI performance using newly developed whitening techniques based on Gram–Schmidt orthogonalization. Whitening transform aids in providing stronger data correlation and unit variance [16]. In the BCICW scheme, the whitening process is as follows:

Step 1: Let *X* be a BCI potential vector of zero-mean data. Then, its covariance matrix is expressed as below:

$$A=Cov\;\left[X,X\right]=E\left[\left(XX\right)\right]=\sum^{\;}\frac{\left(XX\right)}{n}$$

where *X* is an unknown BCI variable, *Cov* [*X*, *X*] or the matrix *A* is the covariance matrix of *X*, and *n* is the number of BCI data. If the data points in *X* are correlated, then their covariance *A*, will not be a diagonal or identity matrix.

Step 2: To de-correlate the data, we need to transform it so that the transformed data will have a diagonal covariance matrix. This transform can be found by solving the eigenvalue problem. We find the eigenvectors and associated eigenvalues of the matrix *A* by solving

AP=PΛ

*Λ* is a diagonal matrix having the eigenvalues as its diagonal elements and the matrix *P* is obtained by taking Gram–Schmidt orthogonalization to the derived eigenvectors. Thus, the matrix *P* diagonalizes the covariance matrix of *X*. The columns of the matrix *P* are the eigenvectors of the covariance matrix. We can also write the diagonalized covariance as (diagonalization or similarity transformation):

(2) $$P^{T}AP=\Lambda$$

If we wish to apply this diagonalizing transform to a single BCI vector of data, we just form: $y=P^{T}X$. Thus, the data *y* has been decorrelated: its covariance, *E*(*yy^T^*) is now a diagonal matrix *Λ*.

$$E\left(yy^{T}\right)=E\left(P^{T}XXP\right)=E\left(P^{T}AP\right)=\Lambda$$

Step 3: The diagonal elements (eigenvalues) in *Λ* may be the same or different. If we make them all the same, then this is called whitening the data. Because each eigenvalue determines the length of its associated eigenvector, the covariance will correspond to an ellipse when the data is not whitened, and to a sphere (having all dimensions the same length, or uniform) when the data is whitened. Whitening is verified as below: $\Lambda^{-1/2}\Lambda \Lambda^{-1/2}\;$= *I*. Equivalently, substituting in Equation (2), we can write: $\Lambda^{-1/2}P^{T}AP\Lambda^{-1/2}$ = *I*. To apply this whitening transform to *y*, we simply multiply it by this scale factor, obtaining the whitened data *w*:

$$X_{whiten}=w=\Lambda^{-1/2}y=\Lambda^{-1/2}P^{T}X.$$

where *Λ* is the eigenvalue, and *P* is the eigenvector of the covariance matrix, and *X* is the BCI data.

Now the covariance of *w* is not only diagonal but also uniform (whitened) because of the covariance of *w.* Thus, we verify the following equation of *E*(*ww^T^*) = *I* as below.

$$E\left(ww^{T}\right)=E\left(\Lambda^{-1/2}P^{T}XX^{T}P\Lambda^{-1/2}\right)=E\left(\Lambda^{-1/2}P^{T}AP\Lambda^{-1/2}\right)=I.$$

This is the whitening process in BCICW.

## 2. Materials and Methods

**Figure 2:**
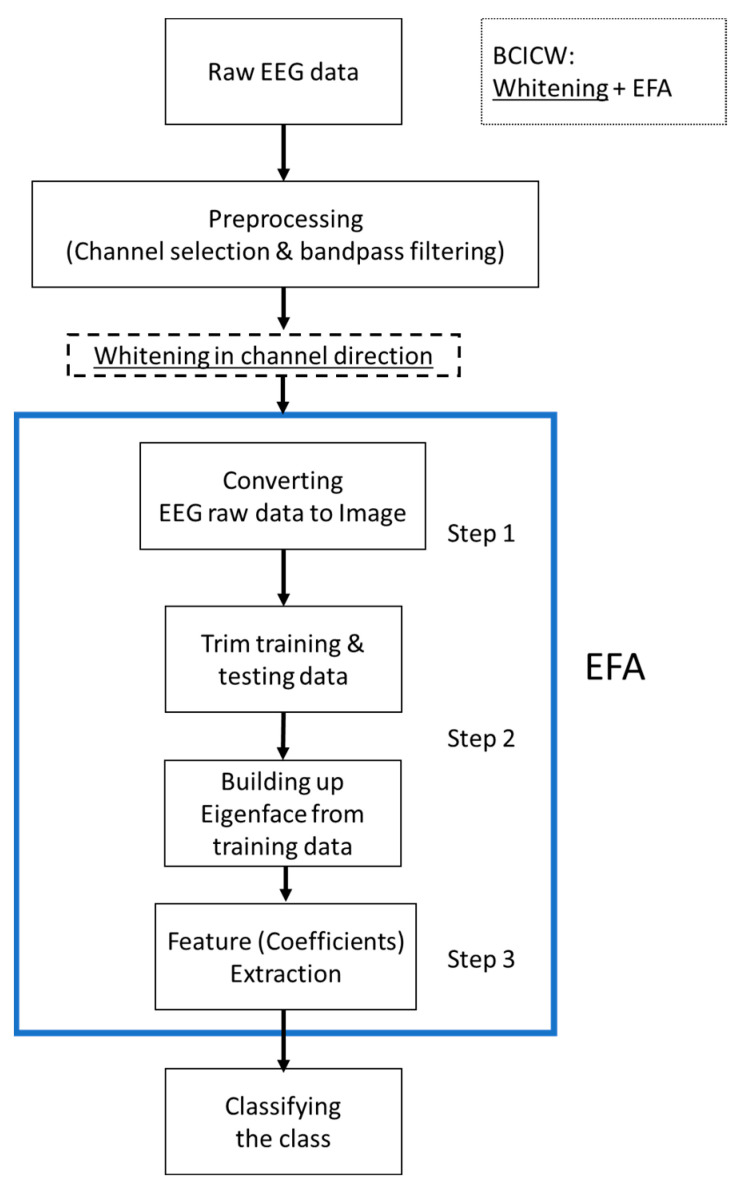
EFA algorithm procedure related to BCICW.

Eigenface analysis (EFA) is a type of PCA that is mainly used to reduce the dimensions in image recognition, particularly for face recognition [16,17,18,19]. In one hand, PCA is the process of calculating the main components and using them to obtain maximum variance axes on the BCI dataset. On the other hand, EFA extracts the featuring images or faces which prioritizes the maximum likelihood on the BCI dataset. Figure 2 depicts the EFA algorithm procedure. To be specific, the mathematical calculation for the EFA method is described as follows into steps 1, 2, and 3:

Step 1: In the first step, the EEG data are converted into image data. The three-dimensional (3D) EEG data can be represented as M time, N channels, and L trials, as described in Equation (3). Therefore, the EEG data can be analyzed with three directions because they form a type of 3D image, and the generated image may differ according to the data viewpoint direction, as illustrated in Figure 2.

Step 2: For the derived image data, the covariance matrix can be obtained. For the given covariance, we determined the eigenfaces. Hence, building up the eigenfaces for the image data has finished.

Step 3: For the given eigenfaces, we can project the training data and thus obtain the results in the features or coefficients for training data. In sequence, projecting the testing data provides the features (coefficients) for testing data. These two types of coefficients are the requested features.

**Figure 3:**
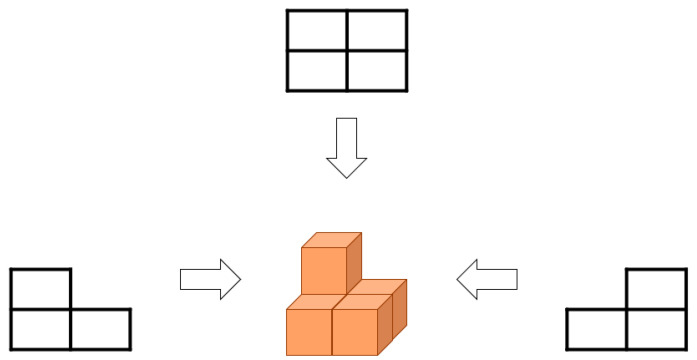
Data analysis depending on the interpretation viewpoint direction.

The mathematical calculation for the EFA method is described as follows: In the first step, the EEG data are converted into image data. The three-dimensional (3D) EEG data can be represented as M time, N channels, and L trials, as described in Equation (3). Therefore, the EEG data can be analyzed with three directions because they form a type of 3D image, and the generated image may differ according to the data viewpoint direction, as illustrated in Figure 3 where the viewpoints in interpretation are top, left side, and right side. As did in MI classification problems for the BCIs [24,25], the tentative datasets *M*, *N*, and *L* are composed of random sample functions, conceptual electro potentials, and the number of trials, and thus, they have no physical units in statistical sense; in fact, those datasets will be coefficients of eigenfaces and part of weighting variables. Subsequently, we built the *M*, *N*, and *L* datasets using those derived coefficients as shown in Figure 2. The different images that are interpreted in different directions for the EEG data also exhibit different analysis results, and it is necessary to select an analysis direction that is suitable for the purpose.

*I* = *MNL* (3)

The original EFA method interprets the EEG image based on the channel. The EEG data in the *MNL* direction are converted into the image dataset I, which is an N image group for each channel in the same manner as that indicated in Equation (4). The image dataset I that is converted from the EEG data consists of N images with *ML* pixels or N vectors with the *ML* direction.

*I* = *M′N* (*M′* = *ML*) (4)

In the second step, the eigenface is built from the converted image, and the image *Φ* with the average value *Ψ* removed is calculated for the N channel image dataset I.

*Φ_i_* = *I_i_* – *Ψ_i_*, *i* = 1, 2, …, *N* (5)

Subsequently, the covariance matrix using the image with the mean removed is computed, as indicated in Equation (6).

(6) $$C=\frac{1}{L}\;\overset{L}{\underset{l=1}{\sum }}\Phi_{i}\Phi_{i}^{T}$$

We define the eigenvectors of *X* and associated eigenvalues of *l* of the covariance matrix *C* by solving

CX=λX

Among the basis vectors that are obtained from this covariance matrix, the k basis vectors that are selected according to the eigenvalue size are known as the eigenfaces *Γ* (*Γ*_1_, *Γ*_2_, …, *Γ_k_*). In this case, the number of vectors k may be selected considering the calculation amount and required data range. The eigenface is used to extract the training and testing features or coefficients [8]. The eigenface created with only training data is defined as the training eigenface *Γ_training_*. In the final step, the training features can be extracted using the training eigenface and training data. Under the supervisor learning model, on this phase, the training features will be associated with the given train labels. The test features can be extracted using the same eigenface and test data. The extraction of the eigenface coefficients is carried out through the data being projected into eigenface space, as indicated in Equation (7).

*Ω_training_* = *Φ_training_ Γ_training_* (7)

The weight coefficient *Ω_training_* that is extracted through Equation (7) is used as a training feature for the data classification. The feature coefficients *Ω_testing_* can be extracted by projecting the test data onto the eigenspace that is trained by the training data, as shown in Equation (8). After training the classifier using the extracted training features, the left/right MI EEG of the test data can be classified.

*Ω_testing_* = *Φ_testing_ Γ_training_* (8)

However, considering a statistical signal processing in an actual and practical BCI system, the application interface is manipulated according to each trial in which the intentional thought of the user is expressed. As the EEG data are 3D data composed of the time, channel, and trial, different images and features are extracted depending on the viewpoints (axes in the coordinate system) or the direction in which the data are interpreted as depicted in Figure 3. If the analysis is performed according to an axis or dimension other than the interpretation of the trials, completely different results may appear in the accuracy classification. If the direction of the image interpretation is changed for the trial interpretation, the source data *I* in the form of *M* × *N* × *L* are reconstructed in the first step of the EFA in Equation (9). However, when the image is interpreted with respect to the trial direction, the EFA accuracy decreased.

*I* = *M′L* (*M′* = *MN*) (9)

According to Reference [26], when the EFA is interpreted in the direction of the trial, the EFA method yields 52.22%, 46.67%, and 63.33% for the three subjects with the same data. Table 1 presents the accuracy when analyzing the trial direction using the EFA method.

**Figure 4:**
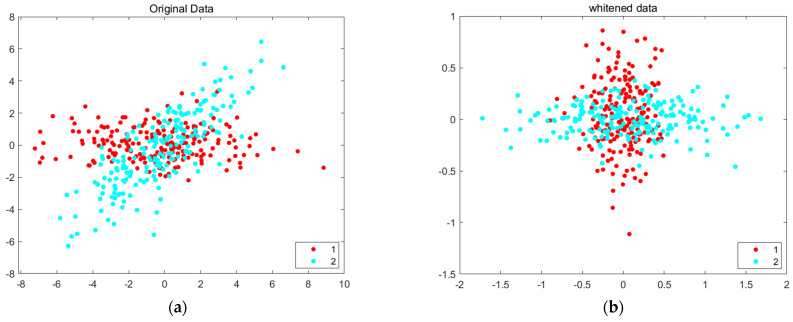
(**a**) Original data and (**b**) whitened data.

Whitening does not perform dimension reduction because it is dependent on PCA. It basically provides a channel independence statistically in the BCI data. Figure 4 presents an example to demonstrate the whitening effect for a certain general data shape. The Gram–Schmidt scheme is for orthogonalizing the vectors and determining the orthonormal basis. For vectors $v_{1},\;v_{2},\;\ldots v_{k}$, orthonormal (orthogonal and normal) vectors $u_{1},\;u_{2},\;\ldots u_{k}$ are calculated using Gram–Schmidt orthogonalization in Equation (10). In Gram–Schmidt, each vector is divided into two components such as tangential and normal components. The normal component is obtained by projecting the vector *v_k_* to a lower vector space *v_i_* or *v_k-1_*, i.e., $proj_{u_{i}}(v_{k})$ which is a tangential component and then computing its residual $v_{k}-\overset{k-1}{\underset{l=1}{\sum }}proj_{u_{i}}(v_{k})$.

(10) $$u_{1}=v_{1}\;u_{2}=v_{2}-proj_{u_{1}}(v_{2})\;u_{k}=v_{k}-\overset{k-1}{\underset{l=1}{\sum }}proj_{u_{k-1}}(v_{k})=u_{k}=v_{k}-\overset{k-1}{\underset{l=1}{\sum }}proj_{u_{i}}(v_{k})$$

Orthonormal (orthogonalized and normalized) vectors $u_{1},\;u_{2},\;\ldots ,\;u_{k}$ are orthogonal to one another, become the orthogonal basis for the vector space, and are then normalized.

In the BCI system, every researcher uses the feature instead of raw data because the raw data is extremely large [7]. Therefore, we cannot use the random data in BCI systems. In terms of computational amount and performance improvement, especially in pattern recognition, the result obtained by eigenvector is not fundamentally orthogonal, so Gram–Schmidt orthogonalization is needed because the covariance matrix obtained from the feature is not symmetric. In the BCI system, the EFA algorithm is a fundamental feature extraction method, and the feature is a determine tools including accuracy [27]. Likewise, in the other reference paper [27,28], they utilize the accuracy in BCI problems using CSP.

## 3. Results and Discussion

The background on EEG datasets from BCI competition for evaluation needs to be explained. To validate and verify the proposed BCICW, we used EEG raw data from three subjects, from the worldwide available and approved off-line datasets of BCI competitions [29]. The datasets contain MI EEG real signals which are recorded as subjects imagine arm or limb movements (e.g., 2 classes for left hand or right hand movements) [10].

The dataset IIIa, BCI competition III (D3D3a) comprises EEG signals from three subjects who performed left hand, right hand, foot, and tongue MI. The EEG electro-potential signals were recorded using 60 electrodes of 10-20 systems. For the purpose of this study, only EEG signals corresponding to left and right hand MI were used [3]. A training and testing set were available for each subject. Both datasets contain 45 trials per class for subject 1, and 30 trials per class for subjects 2 and 3.

For feature extraction, we adapted the EFA method [26], and for classification, we considered the LDA discrete classification of the trials, i.e., we assigned a class to each trial. For each dataset and trial, from raw brain data of BCI competition dataset, we extracted features of EFA from the time segment located from 0.5 s to 2.5 s after the screen cue instructing the subject to perform and imagine MI. Each trial was band-pass filtered in 8–30 Hz considering Brodmann areas as in [18], where a 5th order Butterworth filter is applied [18].

**Figure 5:**
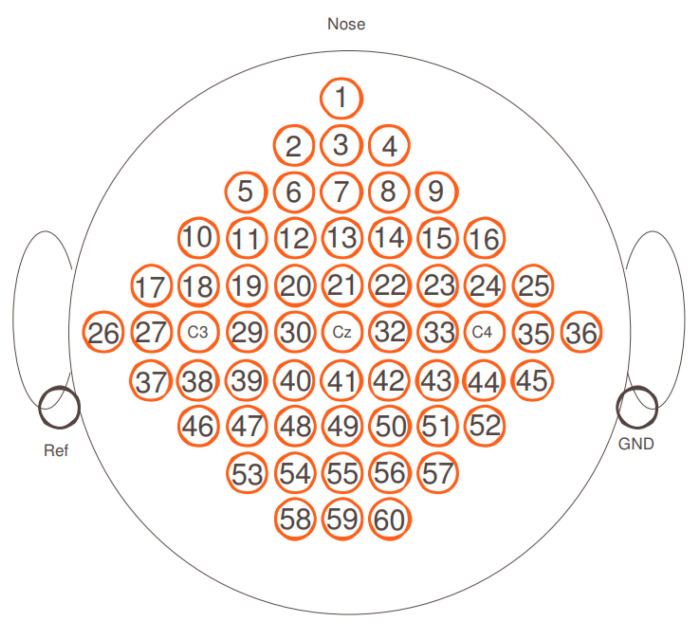
Electrode positions in BCI Competition III dataset IIIa (D3D3a).

This section presents the performance evaluation of the experiments when using the developed BCICW based on the Gram–Schmidt orthogonalization method. The MATLAB program was used for the simulation. The main experiment used the BCI competition III dataset IIIa (C3D3a). The simulated results when using the EFA and the whitening following the EFA methods are compared to verify the accuracy improvement of the proposed method using the data mentioned above. In the experiment for performance evaluation, the MI classification dataset from C3D3a was used to compare and analyze the performances using the same dataset. The C3D3a dataset consists of EEG data for multi-class MI classification. The EEG data were recorded by MI with four classes, namely, the left and right hands, foot, and tongue of three subjects, and were measured using 60 channels from three subjects. Among the four-class data, we considered only two classes: the left- and right-hand classes. Moreover, the left mastoid was used as a reference, and the right mastoid was used as the ground. The EEG data were sampled at 250 Hz and filtered in the range of 1 to 5 Hz through a notch filter. Figure 5 depicts the positions of the EEG electrodes used.

In this experiment, two classes were classified in the feature extraction for the MI classification; thus, it was assumed that there were two characteristics when extracting the data features. When constructing an eigenface, only two basic vectors with the largest corresponding eigenvalues among the basic vectors are used for dimension reduction and noise removal. The most widely applied classification accuracy was used to measure the performance of the MI classification.

An LDA classifier was used for the classification because LDA is one of the most widely used classification methods, and the accuracy was calculated by comparing the class that was predicted by the classifier with the actual class of the corresponding data. Table 2 displays the criteria for the correct answers and errors classified by comparing the predicted and actual labels for the left and right hands. “A, correct” is the classification predicted by the left hand for the actual left-hand data. “B, incorrect” is the classification predicted by the left hand for the actual right hand. “C, incorrect” is the classification predicted by the right hand for the actual left hand. Finally, “D, correct” is the classification predicted by the right hand for the actual right-hand data; therefore, it is determined as the correct classification.

In Table 2, the probability of making a type I error or false alarming is denoted by the letter C and the probability of making a type II error or missing the target is denoted by B. The accuracy is the ratio of the total number of classifications to the number of correct classifications among all classified data, as indicated in Equation (11).

(11) $$A_{cc}=\frac{A,\mathrm{correct}+D,\mathrm{correct}}{A,\mathrm{correct}+B,\mathrm{incorrect}+C,\mathrm{incorrect}+D,\mathrm{correct}}$$

On each trial, we obtained accuracy for each subject, thus the accuracy could be a random variable in statistical senses. On these accuracy values, the variance of accuracy is a measure of dispersion or degree of spreading; indicating the measure of how far or close a set of each accuracy is spread out from the mean accuracy value. 

In the variance comparison and contrasting with the results of EFA among available BCI competition dataset, we used the BCI competition III data set IIIa (C3D3a_2C). Between the BCI competition III data set IIIa (C3D3a_2C) and competition VI data set IIa (C4D2a_2C) for 2 class dataset, we focused on the C3D3a_2C. C3D3a_2C dataset composed of three subjects and the predefined number of experimental trials. Table 3 shows the number of trials per subjects for C3D3a_2C used in this article.

Table 4 presents the results of classifying the MI of BCI C3D3a_2C using only EFA and using BCICW. Compared to the EFA method, the BCICW method improved the variance of the accuracy from 55.00 to 58.15 and dramatically minimized the variance of the accuracy performance among subjects from 58.33 to 11.21; that is, all three subjects exhibited uniform or consistent accuracy when BCICW was applied. Without whitening, a sample output of testing results for C3D3a_2C is given. As a comparison, with whitening a sample output of testing results for C3D3a_2C is given. As shown Box 1, Box 2 and Box 3, from the two outputs, BCICW reduces the variance among subjects dramatically and thus minimizes the discrepancy between existing BCI experiment participants.

Box 1 **A sample output of EFA testing results for C3D3a_2C.**

% EFA primitive classic mode (Whon=0 & EFA_c=1)

dataset: C3D3a_2C

subject 1 : acc 53.333333

subject 2 : acc 48.333333

subject 3 : acc 63.333333

mean 55.00, median 53.33, variance 58.33

Box 2 **A sample output of BCICW testing results for C3D3a_2C.**

% Whitening classic mode (Whon=1 & EFA_c=1)

datsset: C3D3a_2C

subject 1 : acc 57.777778

subject 2 : acc 55.000000

subject 3 : acc 61.666667

mean 58.15, median 57.78, variance 11.21

Box 3 **The detailed information of the property for C3D3a_2C.**

coment1: ‘ dataset: C3D3a_2C’

date: ‘ 2021.12.28 ‘

madeby: ‘ 2C ‘

affiliation: ‘ KNIT ‘

window: ‘ offset : 3.500000e+00, length : 2 ‘

subject: ‘ subject #: 1,2,3’

prefiltering: ‘ off ‘

s: 250 (# of samples/sec)

c: [1]

x: [500 × 60 × 180 double]

y: [1 × 180 double]

**Figure 6:**
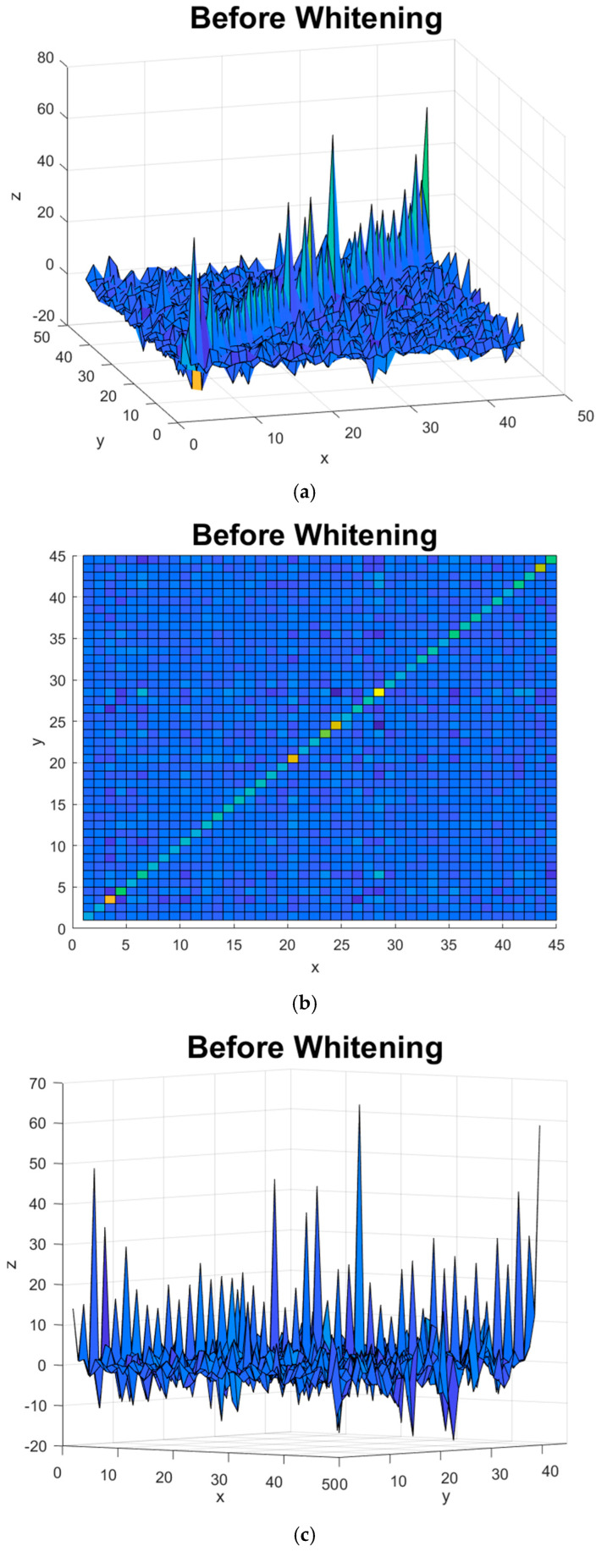

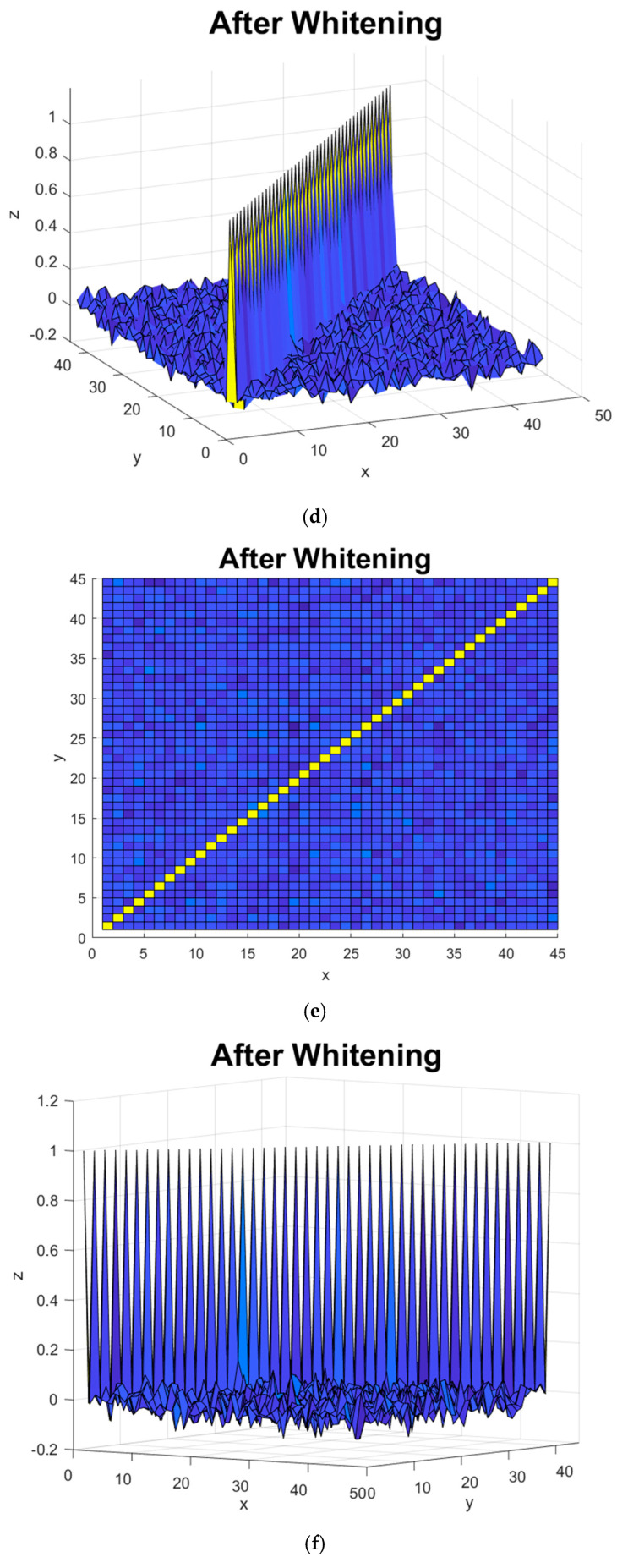
(**a**,**b**) Covariance matrix variances of C3D3a_2C for the first subject without BCICW method; (**c**) enlarged figure of (**a**,**b**); (**d**,**e**) covariance matrix variances of C3D3a_2C without whitening method; (**f**) enlarged figure of (**d**,**e**).

Figure 6a,b present the covariance matrix of C3D3a_2C for the first subject when the EFA method was applied. Figure 6c,d depict the covariance matrix of C3D3a_2C for the first subject when BCICW was applied.

To validate and verify BCICW in a real dataset with a comparison to C3D3a_2C, the next section is for the result of C4D2a_2C. Table 5 shows the number of trials per subjects for C4D2a_2C used in this article. The C4D2a_2C dataset is composed of nine subjects and the predefined number of experimental trials.

Table 6 presents the results of classifying the MI of C4D2a_2C using EFA and using BCICW. Compared to the EFA method, the BCICW method improved the variance of the accuracy from 52.55 to 55.02 and reduced the variance of the accuracy performance among subjects from 17.48 to 9.38; that is, all three subjects exhibited uniform or consistent accuracy when BCICW was applied. Without whitening a sample output of testing results for BCI C4D2a_2C is given. From the given data, the whitening of a sample output of testing results for C4D2a_2C is given. As shown Box 4 and Box 5, from the two outputs, BCICW reduces the variance among subjects significantly, thus minimizing the discrepancy existing between BCI experiment participants.

Box 4 **A sample output of EFA testing results for C4D2a_2C.**

% EFA primitive classic mode (Whon=0 & EFA_c=1)

dataset: C4D2a_2C

subject 1 : acc 53.472222

subject 2 : acc 52.083333

subject 3 : acc 55.555556

subject 4 : acc 55.555556

subject 5 : acc 54.166667

subject 6 : acc 45.138889

subject 7 : acc 58.333333

subject 8 : acc 47.222222

subject 9 : acc 51.388889

mean 52.55, median 53.47, variance 17.48

Box 5 **A sample output of BCICW testing results for C4D2a_2C.**

% Whitening classic mode (Whon=1 & EFA_c=1)

dataset: C4D2a_2C

subject 1 : acc 52.083333

subject 2 : acc 50.694444

subject 3 : acc 52.083333

subject 4 : acc 58.333333

subject 5 : acc 55.555556

subject 6 : acc 59.027778

subject 7 : acc 58.333333

subject 8 : acc 54.166667

subject 9 : acc 54.861111

mean 55.02, median 54.86, variance 9.38

**Figure 7:**
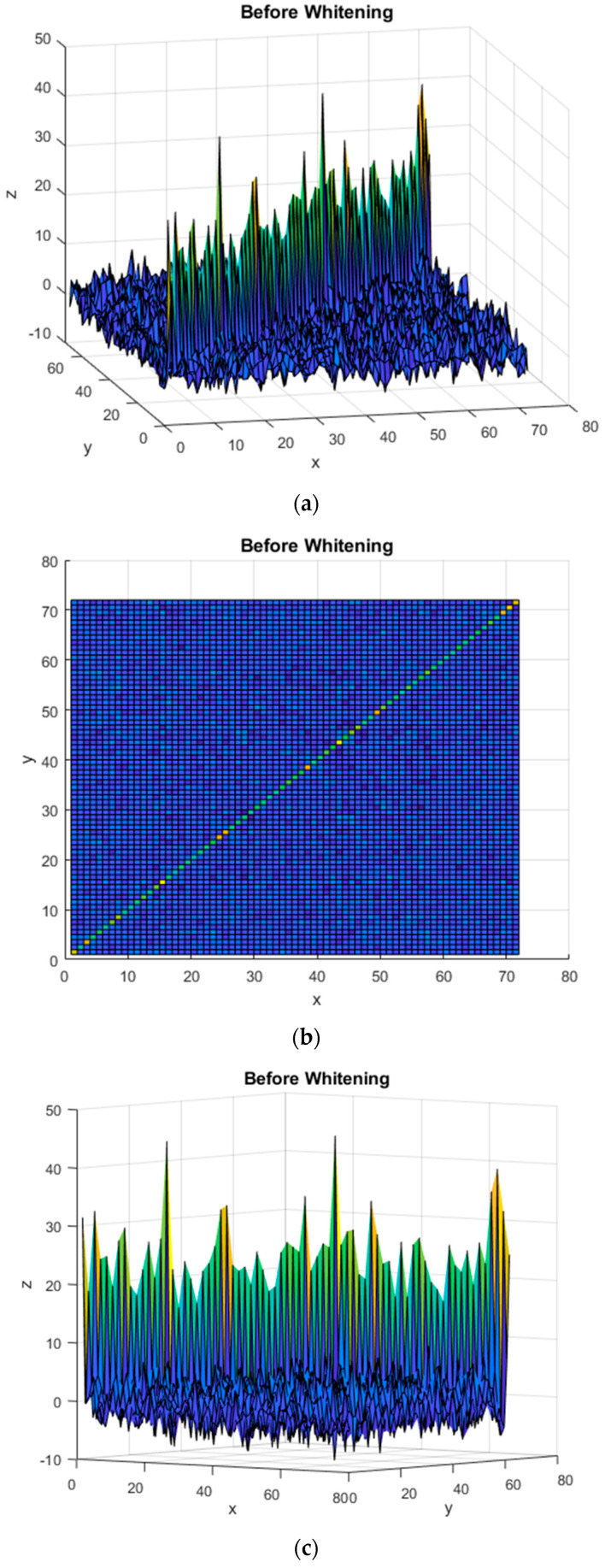

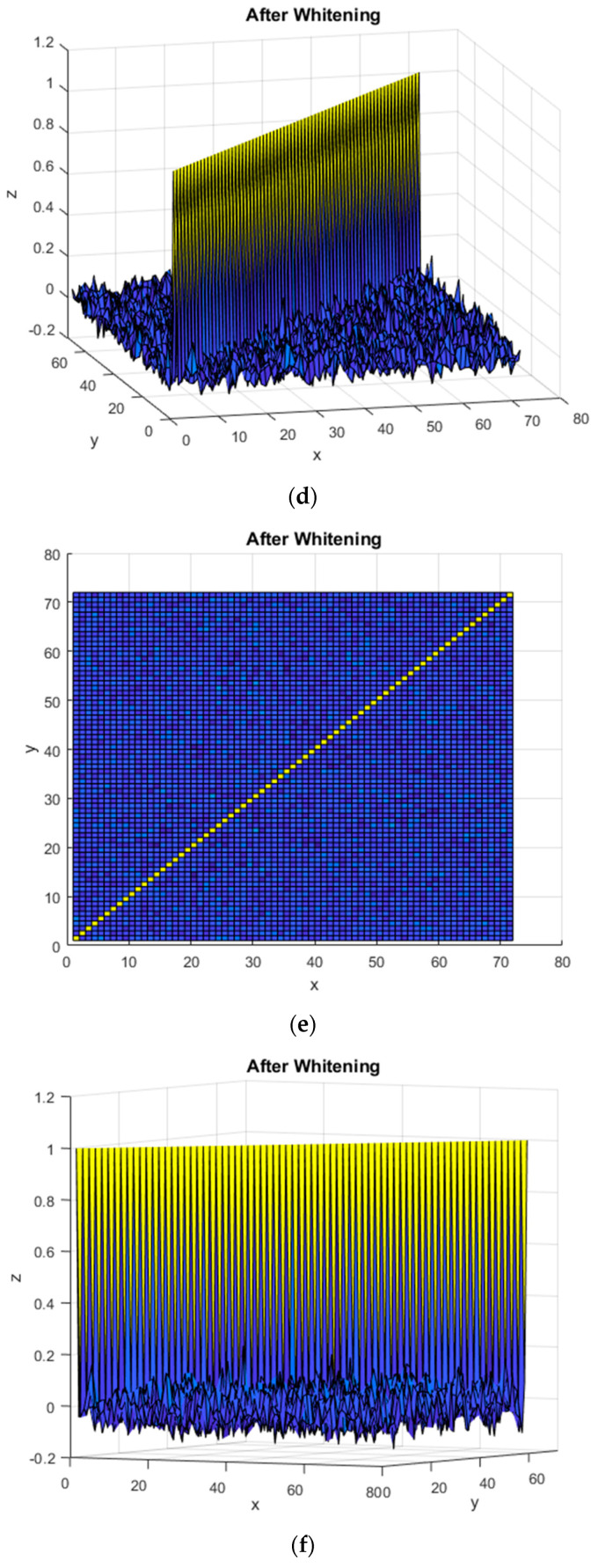
(**a**,**b**) Covariance matrix variances of C4D2a_2C for the first subject without BCICW method; (**c**) enlarged figure of (**a**,**b**); (**d**,**e**) covariance matrix variances of C4D2a_2C for the first subject without whitening method; (**f**) enlarged figure of (**d**,**e**).

Figure 7a,b present the covariance matrix of C4D2a_2C for the first subject when the EFA method was applied. Figure 7c,d depict the covariance matrix of C4D2a_2C for the first subject when BCICW was applied.

Figure 6b and Figure 7b shows the diagonal component of the covariance matrix before BCICW, and the color of the diagonal component is varied because of non-unity. In contrast to this, Figure 6e and Figure 7e show the diagonal component of the covariance matrix after BCICW, and the color of the diagonal component is monotone because of unity. The monotonic color in the diagonal component of the covariance matrix is a key improvement for obtaining the feature extraction for the BCI dataset.

In handling or manipulating covariance matrices, there are two kinds of components such as a diagonal component and an off-diagonal component. The diagonal terms refer to the variance or auto-correlation, and the off-diagonal terms represent cross variance or cross-correlation. From Figure 6, we observed the covariance of BCI data in channel direction is not diagonal, and thus, the measured data on each channel affected each different channel. That is the phenomenon of channel dependence in 10–20 systems. Based on this motivation, we tried to minimize channel dependence among the measured data in electrodes by maximizing the diagonal terms to unity and minimizing the off-diagonal terms, i.e., whitening the data. In fact, the covariance matrix indicates the correlation between data; however, the variance of each trial data is not the same, as is the case with the diagonal components of the covariance matrix. Therefore, a problem occurs that the weight of data with a large variance is simply increased when whitening is performed. Because the whitening method for the channel causes the variance of each trial data to be unity, the variance of all trial data is unity all the same.

Because of whitening in channel direction, the independent eigenface for each class is unique and distinguishable. In addition, the Euclidean distance between the coefficients of left and right classes has been increased. Those contributions result in improved accuracy and a reduced variance. 

## 4. Conclusions

The main purpose of this study was to demonstrate an improvement in the accuracy variance when using the BCICW technique for MI classification. This technique can improve the accuracy for MI classification of BCI systems. Specifically, this study aimed to improve the classification accuracy variance when systematically analyzing and revising the EFA with whitening methods, which process EEG signals as neuro images according to each trial. In the MI classification problem, which is a representative problem for EEG data classification, unlike the common spatial pattern method (CSP), which was mainly used in existing studies, the BCICW method considers signals as whitening-sense neuro images so that it is possible to extend it to classify more than two classes. 

However, in the statistical signal processing framework for EEG, signal data exhibit different and time-varying characteristics depending on the viewpoint of the direction in which the data are interpreted because EEG signal data are 3D data composed of time, channel, and trial. To solve this problem, a whitening method was proposed to guarantee the channel independence for the channel data of the source signal in the feature extraction process from the cooperating EFA method. In BCI classification problems, the accuracy variance among participant subjects is an indispensable and crucial consideration to minimize unfairness issues between subjects.

When analyzing and evaluating each attempt for the BCI implementations, the outcome was that for C3D3a_2C, accuracy variances of 58.33 and 11.21 without and with BCICW, respectively, were recorded; for C4D2a_2C, accuracy variances of 17.48 and 9.38 without and with BCICW, respectively, were recorded, which demonstrates a dramatic decrease in the accuracy variance. In fact, the EEG data for the study of the MI classification problem are the data from three subjects of the C3D3a and the nine subjects of C4D2a_2C, which was used in previous related studies. Therefore, our proposed BCICW technique based on Gram–Schmidt orthogonalization could be effective in reducing the variance for MI classification of BCI applications and provides a constructive testing framework for BCI classification problems.

## Figures and Tables

**Table 1:** Accuracy results of trial EFA.

	Subject 1	Subject 2	Subject 3
Accuracy	52.22	46.67	63.33

**Table 2:** Comparison of predicted and true classifications for left and right hands.

	True Label	
	Class 1, Left	Class 2, Right
Class 1, left	A, correct	B, incorrect
Class 2, right	C, incorrect	D, correct

**Table 3:** The C3D3a_2C dataset composed of three subjects and the predefined number of experimental trials.

Subject	Class (# of Trials)	
	Left (L)	Right^®^
1	45	45
2	30	30
3	30	30

**Table 4:** Variance comparison according to classification methods for C3D3a_2C.

		Subjects				
		A1	A2	A3	Mean	Variance
Accuracy	EFA	53.33	48.33	63.33	55.00	58.33
	Whitening	57.78	55.00	61.67	58.15	11.21

**Table 5:** The C4D2a_2C dataset composed of nine subjects and the predefined number of experimental trials.

Subject		1	2	3	4	5	6	7	8	9
Class (# of trials)	Left	72	72	72	72	72	72	72	72	72
	Right	72	72	72	72	72	72	72	72	72

**Table 6:** Variance comparison according to classification methods for C4D2a_2C.

		Subjects										
		1	2	3	4	5	6	7	8	9	Mean	Variance
**Accuracy**	EFA	53.47	52.08	55.55	55.55	54.16	45.13	58.33	47.72	51.38	52.55	17.48
	Whitening	52.08	50.69	52.08	58.33	55.55	59.02	58.33	54.16	54.86	55.02	9.38

## Data Availability

The data presented in this study are included within the article.

## Abbreviations

The following abbreviations are used in this manuscript:

EEG	Electroencephalogram
BCI	Brain–computer interface
BCICW	BCI channel whitening
LDA	Linear discriminant analysis
PCA	Principal component analysis
EFA	Eigenface analysis
C3D3a_2C BCI	competition III data set IIIa for two classes
C4D2a_2C BCI	competition IV data set IIa for two classes
